# Structural Variations of Prions and Prion-like Proteins Associated with Neurodegeneration

**DOI:** 10.3390/cimb46070384

**Published:** 2024-06-26

**Authors:** Carter Sky Christensen, Sean Wang, Wenshu Li, Danyang Yu, Henry James Li

**Affiliations:** School of Arts and Sciences, New York University Shanghai, 567 West Yang Si Road, Shanghai 200122, China; cc6412@nyu.edu (C.S.C.); sw5764@nyu.edu (S.W.); wenshu.li@nyu.edu (W.L.); danyang.yu@nyu.edu (D.Y.)

**Keywords:** prions, neurodegeneration, Alzheimer’s disease, protein homeostasis, aggregation, prion-like proteins, amyloid beta, hyperphosphorylated tau, alpha-synuclein

## Abstract

Neurodegeneration is becoming one of the leading causes of death worldwide as the population expands and grows older. There is a growing desire to understand the mechanisms behind prion proteins as well as the prion-like proteins that make up neurodegenerative diseases (NDs), including Alzheimer’s disease (AD) and Parkinson’s disease (PD). Both amyloid-β (Aβ) and hyperphosphorylated tau (p-tau) proteins behave in ways similar to those of the infectious form of the prion protein, PrP^Sc^, such as aggregating, seeding, and replicating under not yet fully understood mechanisms, thus the designation of prion-like. This review aims to highlight the shared mechanisms between prion-like proteins and prion proteins in the structural variations associated with aggregation and disease development. These mechanisms largely focus on the dysregulation of protein homeostasis, self-replication, and protein aggregation, and this knowledge could contribute to diagnoses and treatments for the given NDs.

## 1. Introduction

Prions are best understood as a misfolding of proteins within the brain that are not exclusive to just one species and can be transmitted between both humans and animals. The associated prion diseases are also called transmissible spongiform encephalopathies (TSEs). The term prion was coined in 1982 by Stanley B. Prusiner as a shortened form of a proteinaceous infectious particle while studying Syrian hamsters [1]. This infectious, misfolded protein then becomes self-propagating in a not yet fully understood manner. Within humans, the most notable prion is PrP^Sc^, which is the infectious form of the PrP^C^ protein. The presentation of prions in humans can be seen in diseases such as Kuru, Creutzfeldt–Jakob Disease, Gerstmann–Straüssler–Scheinker Syndrome (GSS), and Fatal Familial Insomnia (FFI) [2]. However, there are differences in the pathogenesis of each of these diseases, as some may result from infection or sporadic mutation.

The function of the PrP^C^ protein is of interest due to its possibility to shed light on the prion process, including the development of nervous system cells and cell signaling [3]. Also, of growing interest regarding the PrP^C^ protein is its ability to shift phases, and how these phase shifts may then interact with both prion-like and non-prion-like proteins.

With progress in the study of neurodegeneration and the understanding that prions are misfolded proteins that are capable of self-proliferation [4], the definition of prions has evolved to be broader and more widely encompassing. As the average human lifespan increases, age-related non-prion neurodegenerative diseases (NDs) become more prevalent. Naturally, the understanding of NDs also becomes more crucial. The two most common NDs are the incurable Alzheimer’s disease (AD) and Parkinson’s disease (PD). Both AD and PD have a close relationship with their respective prion-like proteins: p-tau and Aꞵ for AD, and alpha-synuclein for PD. As such, a study of prions and the ND-related prion-like proteins will be beneficial to the understanding of the molecular mechanisms behind these diseases.

**Figure 1:**
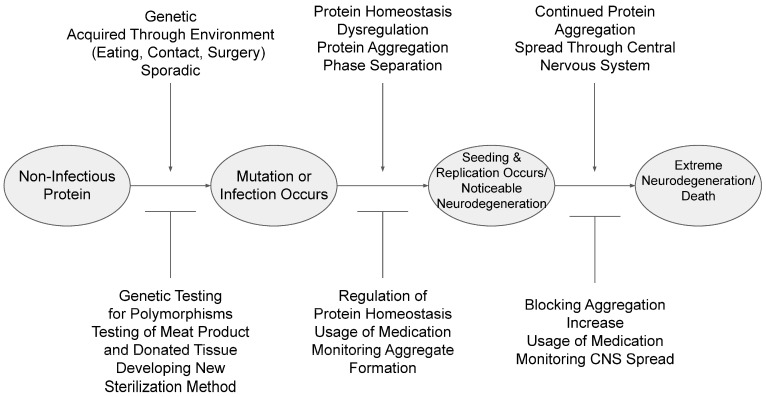
Simplified roadmap of infection progression and potential promoters and inhibitors of infection. There are four main stages represented on the map. Between each is a simplified list of potential promoters (top/arrows) and inhibitors (bottom/flat).

Despite the progress in understanding NDs, the contribution of prions and prion-like proteins in relation to neurodegeneration continues to pose many unresolved questions. One such question is that of the mechanism that regulates ND development and progression. This mechanism can be broken down into three subsections: protein homeostasis (proteostasis), self-replication, and aggregation. As seen in Figure 1 below, these subsections may have both potential promoters (to “aid” in continued neurodegeneration) and inhibitors (to slow/prevent continuation). Furthermore, current research typically focuses on a single ND; however, it may be beneficial to look at different prion-like-protein-related diseases in comparison with each other. As such, this review aims mainly to broadly overview the prion-like proteins associated with ND and summarize the recent advances in the understanding of prions and prion-like proteins through structural variations, underlying regulation mechanisms, and the impact these have on disease-associated protein aggregation.

## 2. Primary Structural Features of Prions and Prion-like Proteins

The four ND-related proteins that this review focuses on are PrP^Sc^, Aβ, p-tau, and alpha-synuclein. While in their biological forms, these four proteins each have their own roles that aid in maintaining neuronal homeostasis, neuronal growth, neuronal repair, and stabilizing neuronal MT. The “infectious” forms become detrimental once a sizable amount has accumulated, allowing for symptoms to be observed.

### 2.1. Prnp, PrP^C^, and PrP^Sc^

**Figure 2:**
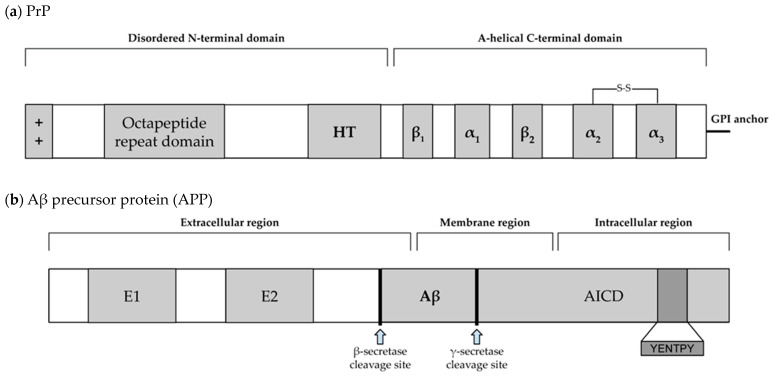

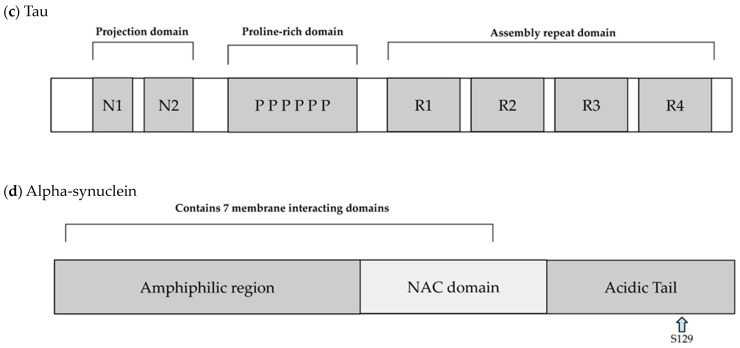
Structures of prion proteins/prion-like proteins. All structures are shown with the N-terminus on the left and the C-terminus on the right. (**a**) shows the general structure of the prion protein; the “++” symbol indicates a positive charge on the N-terminus, HT stands for “hydrophobic tract”; α and β represent alpha-helical and beta-sheet regions, respectively; “S-S” represents a disulfide bridge. Modified from [5]. (**b**) shows the membrane protein APP, as well as relevant cleave sites and relevant end-products; the dark region represents the YENTPY motif in the intracellular region; after this specific set of cleavages, Aβ and AICD will be released into extracellular and intracellular regions, respectively. Modified from [11,12]. (**c**) shows an example of Tau protein; N1 and N2 are exons in the projection domain; R1–4 are imperfect repeats. Modified from [16]. (**d**) represents an example alpha-synuclein protein, consisting of an amphiphilic region, a non-amyglodicemic component domain, and an acidic tail; within the bracketed region, there are 7 membrane interacting domains; the phosphorylation site at serine-129, frequently discussed in the text, is labeled with a blue arrow. Modified from [18].

The 253 amino-acid-long prion protein PrP is encoded by the Prnp gene, which is expressed in the brain and other tissues. The general structure of PrPs can be divided into an N-terminal disordered domain and a C-terminal α-helical domain. The N-terminal domain contains a positively charged region at the N-terminus that is crucial for the endocytosis of PrP^C^, a series of four octapeptide repeats that allow PrP^C^ to bind divalent metal cations (Cu^2^⁺ and Zn^2^⁺), and a hydrophobic tract that is evolutionarily conserved. The C-terminal domain includes three α-helices and two short β-strands. This domain is the site of the post-translational modifications in PrP^C^ with a single disulfide bridge linking helices 2 and 3 (Figure 2a) [5]. The initial non-infectious structural conformation of the prion protein, PrP^C^, is of alpha helices that are attached to the cell membrane at the C-terminal by glycophosphatidylinositol (GPI) [6], and is thought to play a role in cellular adhesion and stem cell renewal [7].

Meanwhile, the infectious form of PrP, PrP^Sc^, is a β-sheet-rich isoform and is highly insoluble, making it difficult to perform a complete structural analysis of it with current methods [8]. There are two main hypotheses as to how the conformational change from the normal, noninfectious PrP^C^ form to the infectious PrP^Sc^ form occurs. The first is that the change is due to the N-terminal of the PrP^C^ protein, which is disordered and thus has a flexible tail. The second is the change that occurs at the C-terminal of the protein, which is speculated to be a highly ordered globular domain containing two antiparallel β-sheets and three α-helices. These distinct differences leave room for speculation as to which terminal may be more in control of form conversion [8,9]. Furthermore, there are distinct strains of the PrP^Sc^ form, which can be categorized by histology, clinical signs, and most commonly, the incubation period (the time elapsed between inoculation and the onset of disease in animal models) [10]. However, even though there are strains that are distinct biologically, not all can be distinguished biochemically [10].

### 2.2. Amyloid and Hyperphosphorylated Tau

The proteins present in AD-associated neurodegeneration are now deemed to be prion-like proteins. This designation results from the replication using a so-called self-template, similar to what is seen in prion proteins. The complete mechanisms behind self-replication are still not understood for both prion-like proteins and prion proteins, but there are many proposed mechanisms that are beginning to be widely accepted.

All amyloid precursor protein (APP) family members share conserved E1 and E2 extracellular domains, an APP intracellular domain (AICD), and the YENPTY motif at the carboxyl terminus (Figure 2b) [11,12]. The APP is a type-I transmembrane protein whose proteolysis gives rise to amyloid-ꞵ peptides. APP processing can undergo one of two pathways: one being the non-amyloidogenic pathway and the other being the amyloidogenic pathway. This second pathway contains what eventually leads to the aggregation of flexible, soluble oligomers that cause neuropathy [13]. In this pathway, alternative cleaving by γ-secretase leads to two notable forms of Aβ: Aβ40 and Aβ42, where Aβ42 contains two additional amino acids [13]. Both Aβ40 and Aβ42 are able to aggregate into fibrils; however, Aβ42 is faster to form aggregates and more neurotoxic [13,14].

Of interest is how exactly the Aβ protein causes memory deficits alongside neurodegeneration. Aꞵ can occur either sporadically or due to genetic factors. However, a recent study demonstrated that human Aβ entering mice’s circulation can induce the occurrence of Aβ plaque in the brain, suggesting that Aβ derived from blood can enter the brain [15]. Aꞵ pathology can be introduced to mice without human Aβ (hAꞵ) by parabiosis. These mice can then develop AD-related pathologies as well as experience phosphorylation of tau. However, what remains to be fully seen in this model is if there is a notable cognitive decline [15].

Tau, as has been mentioned, is an important factor in microtubule (MT) generation and degeneration. Tau consists of three domains, with the projection domain in the amino-terminus, the proline-rich domain in the middle, and the assembly domain in the carboxyl-terminus. The assembly domain contains repeat domains and flanking regions, and supports microtubule assembly (Figure 2c) [16]. As tau undergoes phosphorylation, its binding to the MTs weakens until it dissociates, and then the p-tau will begin to aggregate together. These disordered aggregates of p-tau are known as neurofibrillary tangles (NFTs) and neuropil threads and are one of the hallmarks of AD. While unphosphorylated tau does not significantly aggregate in the absence of artificial inducers (such as heparin), p-tau is able to spontaneously aggregate [17]. In addition, the treatment of neuroblastoma cells with p-tau is sufficient to cause cell death, whereas treatment with non-phosphorylated tau does not significantly affect cell viability [17].

### 2.3. α-Synuclein Proteins

Alpha-synuclein is a protein predominantly found in presynaptic terminals and plays a role in regulating synaptic vesicle function. All α-synuclein proteins comprise three basic domains, including an N-terminal amphipathic region, a central non-amyloid component (NAC) domain, and a C-terminal acidic domain. The seven membrane-interacting amino acid motifs are also in the first half of the protein. The region preceding the NAC domain contains all pathogenic α-Syn mutations identified so far (Figure 2d) [18]. In neurodegenerative disorders such as Parkinson’s disease and related synucleinopathies, alpha-synuclein undergoes conformational changes and forms pathological aggregates. Recent studies have shed light on the structural variations of alpha-synuclein and their association with neurodegeneration.

The elucidation of these novel structural variations of alpha-synuclein provides valuable insights into the mechanisms underlying alpha-synuclein-mediated neurodegeneration. The distinct conformations, strains, and aggregation states of alpha-synuclein may contribute to the diverse clinical presentations and disease progression observed in synucleinopathies. For example, different diseases may have significantly different alpha-synuclein aggregation sites and pS129 levels [19,20]. This, taken together with the observation that pS129 level correlates with disease progression [19], shows promise in the use of pS129 as a biomarker for PD and other related diseases. Moreover, these structural variations offer potential targets for the development of therapeutic strategies aimed at modulating or preventing the formation of toxic alpha-synuclein species.

## 3. Structural Variation of Prions and Prion-like Proteins and Their Association with Neurodegeneration

### 3.1. Structural Variation of Prions and Neurodegeneration

Over the past decade, significant progress has been made in elucidating the unique structural variations of prion proteins and prion-like proteins, including tau, which are intimately linked to the pathogenesis of various neurodegenerative disorders. Prion diseases, such as Creutzfeldt–Jakob disease (CJD), are characterized by the accumulation of misfolded prion protein (PrP^Sc^) in the brain, leading to neuronal dysfunction and ultimately neurodegeneration. Recent studies employing advanced structural biology techniques, such as cryo-electron microscopy (cryo-EM) and X-ray crystallography, have provided unprecedented insights into the three-dimensional architecture of PrP^Sc^ [21,22].

In 1994, a groundbreaking study by Wickner and colleagues, published in the journal Science, provided genetic evidence for the existence of prion-like behavior in the yeast *Saccharomyces cerevisiae* [23]. The genetic demonstration of the prion-like behavior of Sup35 in yeast provided a paradigm-shifting insight into the nature of prion diseases. It established that prion formation and propagation can occur through protein conformational changes rather than solely genetic mutations [23].

One of the most remarkable findings is the existence of distinct strains of PrP^Sc^ with different conformations, which contribute to the diverse clinical phenotypes observed in prion diseases. High-resolution cryo-EM studies have revealed that PrP^Sc^ adopts a beta-sheet-rich amyloid-like structure, distinct from the predominantly alpha-helical structure of its normal cellular isoform (PrP^C^). Furthermore, these studies have demonstrated that PrP^Sc^ can adopt multiple distinct conformations, each associated with a specific prion strain and clinical presentation. The ability of these different conformations to induce strain-specific templated misfolding of PrP^C^ highlights the structural diversity of prions and its direct impact on disease progression [24,25,26,27].

### 3.2. Structural Variation of Prion-like Proteins and Neurodegeneration

In addition to prion diseases, other neurodegenerative disorders, such as Alzheimer’s disease, exhibit prion-like behavior, wherein specific proteins misfold and self-propagate within the brain, leading to the formation of pathological aggregates [28,29]. Tau, a microtubule-associated protein involved in stabilizing neuronal microtubules, is a key player in Alzheimer’s disease and related tauopathies. Recent studies have shed light on the conformational changes that tau undergoes during disease progression. Cryo-EM studies of tau aggregates isolated from human brain samples have revealed filamentous structures comprising twisted paired helical filaments (PHFs) and straight filaments (SFs), which are rich in beta-sheet secondary structures distinct from the predominantly random coil and alpha-helical structure of normal tau [21,30]. Furthermore, the cryo-EM studies have revealed that tau aggregates exhibit polymorphic structures, meaning they can adopt different conformations and morphologies [31]. This structural polymorphism may contribute to the wide spectrum of clinical phenotypes observed in tauopathies, including Alzheimer’s disease, frontotemporal dementia, and progressive supranuclear palsy. As tau undergoes conformational changes, the ability to undergo phosphorylation also changes, along with the ability of a kinase to access a given site on the tau protein [32]. There are strong indications that the tau in AD is resistant to being dephosphorylated due to the presence of filaments within the NFTs [32].

In line with structural variation, recent studies have identified distinct strains of tau aggregates that exhibit variations in their biochemical properties and anatomical distribution [30,33]. These tau strains are reminiscent of prion strains and are associated with different clinical phenotypes and disease progression. The existence of different tau strains and their strain-specific conformations contribute to the heterogeneity observed in tauopathies and provide insights into the mechanisms underlying the spread of tau pathology throughout the brain.

Both Tau and Aβ oligomeric species interact with PrP^C^ to elicit opposite effects on synaptic plasticity, probably mediated by different effectors [34]. These distinct tau strains exhibit variations in their biochemical properties and anatomical distribution, contributing to the diversity of clinical phenotypes. The elucidation of tau’s structural variations and the identification of strain-specific conformations have opened new avenues for therapeutic interventions targeting the pathological conformations of tau [21,33]. These findings highlight the complexity of Aβ/tau pathology and provide potential targets for the development of therapeutic interventions aimed at modulating or targeting specific conformations or strains of Aβ/tau.

Similarly, alpha-synuclein aggregates also exhibit structural polymorphism. Firstly, alpha-synuclein aggregates can be categorized by the degree of fibrillation (unaggregated monomers, short oligomers, proto-fibrils, and fibrils), and pS129 versus unphosphorylated at S129. It is commonly believed that oligomers are the main contributors to toxicity, as the introduction of oligomers can cause alpha-synuclein pathology more than fibrils [35], but fibrils are the end product of aggregation, making it difficult to study oligomers in isolation [35]. Furthermore, there is evidence that alpha-synuclein aggregate conformation may depend on the phosphorylation at pS129, which also affects toxicity [36]. However, exactly which physical properties cause disease pathology is yet to be clearly determined.

One of the key structural variations of alpha-synuclein is its transition from a soluble, predominantly unfolded monomeric state to an insoluble, aggregated state. This transition involves the adoption of beta-sheet-rich conformations by alpha-synuclein, leading to the formation of oligomers, protofibrils, and fibrils [37,38]. Cryo-EM studies have provided high-resolution structural insights into the fibrillar forms of alpha-synuclein. These studies have revealed that alpha-synuclein fibrils adopt a characteristic cross-beta structure, with beta-strands aligned perpendicular to the fibril axis [39].

Parkinson’s disease and other Lewy body diseases are heavily associated with phosphorylated serine 129 (pS129), with >90% of synucleinopathy brains showing significant accumulation of pS129, compared to <4% in normal brains [37]. This finding suggests that alpha-synuclein may be toxic; however, the exact relationship between pS129 of alpha-synuclein and its aggregation is currently unclear. While it might be logical to assume that pS129 functions similarly to hyperphosphorylation of tau and promotes aggregation, it seems not to be the case. In fact, a recent study showed that pS129 decreases both the rate of alpha-synuclein seeding and aggregation [40].

Furthermore, recent studies have highlighted the existence of distinct strains or conformers of alpha-synuclein aggregates. These different strains exhibit variations in their biochemical properties, cellular toxicity, and anatomical distribution [36,41,42]. For example, alpha-synuclein aggregates seem to function differently depending on the seed used: even though unphosphorylated alpha-synuclein forms aggregates at a faster rate (corroborating the finding in [40]), the slower-forming aggregates made by pS129 demonstrated a higher cytotoxicity [36]. The presence of different strains of alpha-synuclein aggregates may contribute to the heterogeneity observed in synucleinopathies, including differences in clinical phenotypes, disease progression, and response to therapeutic interventions.

In addition to fibrillar forms, recent research has also uncovered the significance of soluble oligomeric species of alpha-synuclein in neurodegeneration. These oligomers are thought to be highly toxic and play a crucial role in impairing cellular functions and promoting neuronal dysfunction [43,44]. The structural properties of these oligomers, such as their size, shape, and stability, may influence their toxicity and pathological effects.

## 4. Mechanistic Insights into Structural Changes Driving Protein Aggregation and Neurodegeneration

### 4.1. Dysregulation of Protein Homeostasis Associated with Protein Aggregation

Homeostasis of proteins (proteostasis) is becoming a critical field in ND, as the maintained regulation of protein accumulation can prove crucial for decreasing or negating the likelihood of developing an ND. When proteostasis undergoes dysregulation, the given protein system is ultimately disrupted. It is this dysregulation that then gives rise to the blockade of proteasomal/lysosomal dependent protein degradation and the accumulation of aberrant proteins, as there is no longer a well-maintained level of newly synthesized proteins and a well-maintained clearance of old or damaged proteins.

Impaired proteasomal degradation has been strongly associated with the accumulation of misfolded and aggregated proteins in neurodegenerative diseases such as Alzheimer’s disease and prion diseases [45,46]. In Alzheimer’s disease, the proteasomal system is compromised, leading to the accumulation of amyloid-beta plaques and tau tangles, which are hallmark protein aggregates [47,48,49]. Similarly, in prion diseases, the abnormal conversion of prion proteins into a misfolded and aggregated form overwhelms the proteasomal machinery, resulting in the accumulation of infectious prion particles [50,51]. Therefore, impaired proteasomal degradation plays a crucial role in the pathogenesis of both Alzheimer’s disease and prion diseases by contributing to the formation and persistence of protein aggregates [49].

Within proteostasis, there are many facets to consider for the clearance of overabundant proteins as well as defective proteins that can accumulate to cause pathological issues. As such, the mitochondrial quality of cells within the CNS may play a crucial role in controlling the neuropathology caused by PrP^Sc^, Aβ, and p-tau within these proteins’ respective associated NDs. A recent study found that in cells infected with scrapie, there was an excessive induction of mitophagosome and mitophagolysosome formation, and the PrP^Sc^ protein was enriched in these areas, alluding to the possibility that scrapie infection promotes mitophagy [52].

Impaired brain clearance mechanisms responsible for protein accumulation in NDs provide new diagnostic and therapeutic opportunities to delay or prevent clinical symptoms. The glymphatic system is one such, rather newly discovered, system that may be of significance in regard to the clearance of parenchymal proteins such as Aβ and p-tau seen in AD. As a pathway for exchanging cerebrospinal fluid (CSF) and interstitial fluid (ISF), the glymphatic system is facilitated by aquaporin 4 (AQP4). Harrison et al. found impaired CSF-ISF exchange and AQP4 polarization in a mouse model of tauopathy. A novel drug-based AQP4-specific inhibitor, TGN-020, was shown to decrease tau and p-tau clearance, implicating faulty AQP4 in tau accumulation and suggesting that the glymphatic system could be a druggable target for AD treatment and possibly other NDs as well [53].

Liquid-liquid phase separation (LLPS) is a dynamic and reversible process that assembles biomolecular condensates as well as separates homogenous solutions into different phases: dilute and dense [54]. LLPS within proteins is part of a complex system that helps to control cellular functions, and abnormalities within this can lead to disease development [55]. As such, the PrP protein is not unique in the fact that it undergoes LLPS, having similar characteristics to other proteins that undergo LLPS, such as binding to nucleic acids (NA) and being highly disordered [56]. Therefore an additional mechanism in maintaining protein homeostasis is that of protein phase separation. Understanding the conversion from non-infectious to infectious forms of proteins is of great importance, as doing so creates pathways for potential treatments and diagnostic routes. It is also worth noting that the interaction between PrP^C^ and the amyloid β oligomer (AβO) present in AD can trigger a phase shift in their previously established relationship [57].

The phase separations of the highly disordered PrP (and other ND-causing proteins) can be manipulated by NA aptamers to create aggregates of the bound NA-protein complexes in similar ways to NA-free protein aggregates [56]. As protein concentration increases, the ratio of NA to protein becomes increasingly important to the likelihood of droplet formation and eventual aggregation. The addition of various NA aptamers impacts the protein structure and its ability to aggregate and maintain an aggregated state. Furthermore, there are studies showing that tau also undergoes phase separation and can form complexes with RNA in varying salt concentrations [55]. Therefore, by elucidating the mechanisms by which differing NAs cause conformation changes and aggregation of prion and prion-like proteins into condensates, methods to prevent aggregation may be developed by preventing phase separation dysregulation.

Also, based on the high affinity interaction of PrP^C^ with amyloid-β oligomers (AβO) and the fact that PrP^C^ undergoes an LLPS in which α-helical Thr becomes unfolded, Kostyley et al. found the interaction between PrP^C^ Lys residues and AβO created a hydrogel containing immobile AβO and relatively mobile PrP^C^. NMR studies of hydrogel PrP^C^ revealed a specific α-helical conformation for natively unfolded amino-terminal Gly and Ala residues. Additionally, recombinant PrP^C^ could extract endogenous AβO from human Alzheimer’s brain lysates into hydrogel, and a PrP^C^ antagonist could release AβO from endogenous brain hydrogel [57]. The findings suggest that Aβ species from AD can drive coupled phase and conformational transitions of PrP^C^.

### 4.2. PTM-Associated Structural Anomalies and Protein Aggregation

Alterations in primary gene and/or protein sequences or the formation of new structural domains, including by peptide cleavage and mutations, are essential as the structural basis of protein aggregation. Therefore, the differences in the conformation of a given protein may be a crucial factor in determining if an infection can fully take root in the brain. All three proteins being examined in this review are subject to structural changes that can then result in a distinct strain, possibly resulting from gene mutations or abnormal cleavage of proteins, both of which may be affected by post-translational modification (PTM).

Accounting for factors, such as glycoforms, within a structural model may give rise to better representations of the various strains that are present within prion diseases, since how different clinical phenotypes manifest from different structures of the same protein elicit remains poorly understood. Consideration of structural diversity may be the clue to understanding why these varying strains occur, based on ratios of un-, mono-, and di-glycosylated glycoforms and sialoglycoforms [58].

Regarding the PrP^C^ protein, differences in glycosylation help determine the likelihood of the formation of newly generated PrP^Sc^ proteins and their various associated strains, as well as the ability and patterns of these proteins to deposit within the CNS [59]. Beyond just the glycosylation of the PrP^C^ protein being a source of new strains, patterns of carbohydrate epitopes on N-glycans, sialyation, and glycan charge may also have an impact on strain formation and chances of occurring [60]. Tau is also subject to many different strains based on whether and where it experiences phosphorylation, acetylation, and ubiquitination, which then dictates whether the tau will be able to participate in AD fibrils [60].

While tau proteins may be phosphorylated by many different kinases in the brain, phosphorylation by CaM kinase is what is deemed responsible for the tau in the NFTs witnessed in AD [61]. It is not fully understood what mechanisms cause tau to become hyperphosphorylated, but one speculation is that it occurs due to an impairment of glucose levels, causing decreased tau O-GlcNAcylation, which then causes hyperphosphorylation of tau that eventually leads to NFTs [62]. The NFTs that form eventually accumulate as insoluble forms. In a healthy brain, tau exists intrinsically disordered and can become ordered by having a binding partner. However, tau in an unhealthy brain may be “misdisordered” and then misordered, and this has been detected by the use of the antibody DC11, which binds to the truncated tau proteins in AD [63]. These findings demonstrate the impact of post-translational modification on the transition of structural anomalies.

### 4.3. Regulation of Protein Structures by Environmental Factors: Chaperones, RNA, and Ions

Present in prion diseases, as well as AD, is the development of amyloids, which eventually progresses into amyloidogenesis as neurodegeneration progresses [64,65,66]. There may also be a macroenvironmental dependence that dictates the amyloid formation and infection chances of prion and prion-like proteins [67], as well as the strain formation of the prion protein [68]. Additionally, the microenvironment factors may also help determine the prion protein structure, replication, and toxicity [69]. Along with the environmental dependence, concomitant is the hydrophobicity that may be present within a species due to alterations [70]. Metal ions, such as copper, may also have a role in defining protein conformations. Depending on the region of the protein and the type of ion being considered, the protein may experience a looser or tighter conformational change, as well as affect the likelihood of conversion from the noninfectious form to the infectious form [71].

The chaperones of the proteins can also serve to protect from infectious form conversions, but they can also be catalysts for these infectious forms to occur, depending on their abundance. The different chaperones introduced to a protein may also have different efficiency rates in protection and infection; this can be seen in studies focusing on yeast prions and their varying mutability [72,73]. The presence of RNA in an environment may also impact the conformational changes that a protein may undergo. Rai et al. found that in heterotrophic conditions with RNA present, the proteins of interest are able to undergo transformations until they eventually form a solid-like aggregation [58]. RNA aptamers also serve as a potential way to control the conformational change or structure that may occur, but there is still much to be investigated regarding the use of these aptamers regarding structural regulation.

In sum, the deep molecular basis of prion strains and the factors leading to the formation and propagation of prions is a complex and fascinating area of research. Key insights into the molecular basis and factors involve combinations of prion protein conformation, strain-specific conformations, prion strain diversity, and factors influencing prion formation and propagation.

## 5. Disease-Related Self-Replication and Aggregation Model

### 5.1. Self-Replication of Prions and Prion-like Proteins

In attempts to elucidate self-replication of the prion or prion-like proteins, many models have been created, but seemingly none have fit the disease models perfectly. Elucidating the way prion and prion-like proteins replicate is arguably a key to understanding why these aggregates occur and how to potentially treat and prevent them. The self-replication of these proteins is based on a conformational change of the protein, essentially a refolding of the native protein into a structurally changed molecule. This is seen within the PrP^C^ to PrP^Sc^ conversion, as the protein structure changes from being largely composed of alpha helices to being slargely composed of beta sheets. Additionally, it has been found that all disease-associated proteins have a faster doubling time compared to their relevant biological time scale, further suggesting that replication is an essential mechanism of prion diseases [74]. The difficulty of fully understanding this remains, however, due to the differences that may result from in vitro versus in vivo studies.

The protein aggregation seen in AD is of interest due to the impact of two prion-like proteins at work. The aggregation associated with the Aꞵ plaques is still yet to be fully understood, as it can occur in different structural forms: roughly spherical or irregular wispy deposits [75]. The phosphorylated tau protein aggregates into NFTs that eventually spread throughout the brain [62], in a manner similar to PrP^Sc^ [57]. The spread of infectious proteins via aggregation may also be influenced by the presence of chaperones, as inhibition of chaperone proteins may promote infection and protein aggregation, while enhancement of chaperones may be a potential way to prevent the spread of infectious protein aggregates in a homeostasis-maintaining manner [76,77]. For example, the neuronally expressed chaperone proSAAS can help block Aβ fibrillation [78], and the overexpression of Rnq1 decreases [RNQ^+^] prions toxicity, thus preventing the conversion of other proteins into amyloid species [71].

**Figure 3:**
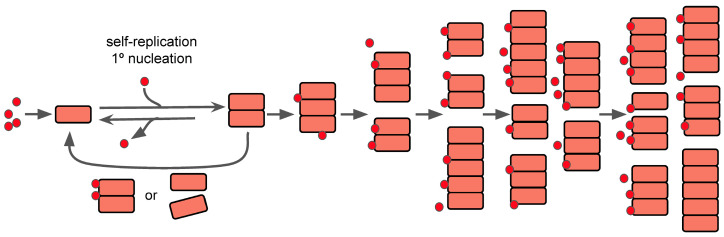
Self-replication and nucleation model of aggregation reactions and concentration over time. The red dots are representative of seeds for nucleation, which are small oligomers of the misfolded prion-like protein. The pink squares represent the aggregates of proteins over time as they clump together to form larger structures. As time progresses, seeding continually happens, and the aggregates can continue to grow in length and/or split to create new ones. Modified from [74].

The simple act of aggregation may have initially had an evolutionary benefit for modern humans, assuming that the protein(s) in question can form filamentous aggregates, as many non-prion-like proteins may form filamentous aggregates and are largely dependent on a secondary nucleation point, as described by Meisl et al. in 2022 [74]. It is this secondary nucleation that causes the divide between normal growth and self-replication, as the secondary process is what allows the system to become autocatalytic and begin to grow exponentially. As seen in Figure 3, the introduction of new protein seeds on the surface of the aggregates allows for the continued growth/lengthening and formation of new aggregates as time goes on. As such, understanding the full function of the protein(s) role in the CNS may serve as a way to begin to focus studies about preventing aggregation or other secondary processes.

### 5.2. Neurological Inflammation

Alongside the protein self-replication and aggregation mechanisms, another factor that may influence the mechanistic progression of NDs is that of neurological inflammation. Initially, neuroinflammation may serve a protective role within the CNS, but if it is prolonged or under dysregulation, it may begin to aid in the progression of diseases [78]. There is also growing evidence that neurological inflammation may serve as a way to spread infectious proteins throughout the CNS via astrocytes and microglia [79,80].

Regarding neurodegeneration caused by both prion and prion-like diseases, the potential role that astrocytes may play in the spreading of these misfolded proteins is of interest. It has been noted that animals have aggregates in astrocytes prior to the appearance of disease symptoms [81], heavily suggesting that astrocytes play a major role in prion infection [82]. As animal-based prions act rather similarly in infection models, it is possible to speculate that the human astrocyte would also be playing a crucial role in both prion and prion-like infections. In a PS19 mouse model, as early as 3–4 months, the microglia are activated in the brain and spine, and at 6 months old, there are reactive astrocytes in the CA3 region of the brain in a study focusing on tauopathy [83]. This hypothesis has been further studied in animal models, as has the role of astrocytes in neuroinflammation in not only prion disease but prion-like protein diseases, such as AD. Worth noting, however, is that in astrocyte-associated infection and microglia-associated infection by extension, spreading may be limited to a specific type of infection due to there being three categories: genetic, sporadic, and acquired, which occur at different rates.

## 6. Perspectives in Diagnosis, Prevention, and Therapy

Recent studies have shown that there may be an interplay between astrocytes and glial cells, which could potentially be beneficial in reducing infectious forms but, at the same time, may also contribute to the spread of infectious proteins in the central nervous system (CNS) [80]. Currently, there are no tests that can be performed on a still-living individual that can conclusively diagnose an individual with an ND, but there are tests that can be performed that have a high level of confidence regarding the given diagnosis, such as magnetic resonance imaging (MRI), positron emission tomography (PET), computed tomography (CT), single-photon emission computed tomography (SPECT), spinal tap to sample spinal fluids, and electroencephalograms [84]; however, the only truly definitive way to diagnose NDs is post-mortem during a brain autopsy (neuropathology autopsy).

All present methods of treating NDs are aimed at halting the progression of symptoms while also trying to prevent any new symptoms from developing for as long as possible. As of now, there is no cure for any ND, but there is considerable interest in developing increasingly improved treatments until a cure can be developed.

Both pharmaceutical-based and therapeutic-based treatments have a large number of potential targets, such as (1) the prevention of Aβ protein from forming, accumulating, and spreading within the CNS; (2) delaying and preventing cognitive decline from occurring and treating it when/if it does occur; and (3) treating neuroinflammation and any resulting apoptosis while trying to enhance neuroprotection. Treatments targeted towards the p-tau protein need to focus on the reduction of hyperphosphorylation from occurring and a reduction of the accumulation and spread of p-tau and NFTs in the CNS, as well as a similar focus as (2) and (3) in the treatment of the Aβ proteins. Similar to treatments for Aβ protein and p-tau protein, the treatments for the PrP^Sc^ protein will also need to focus on prevention of formation, accumulation, and spread, as well as the previously listed focuses of (2) and (3), but also need a focus on preventing infection from being able to potentially spread to other individuals and species.

### 6.1. Pharmaceutical-Based and Therapeutic-Based Treatment Methods

Pharmaceutical-based treatments for neurodegeneration have largely focused on immunosuppression in order to prevent the large spread and continual occurrence of misfolded proteins. Currently, pharmaceutical treatments largely focus on researching the repurposing of existing medications [85].

Therapeutic treatments for neurodegeneration are largely focused on individuals who have a genetic predisposition to develop an ND. This focus aims to delay the onset of noticeable symptoms for as long as possible and tends to rely on pharmacological approaches in order to stave off the accumulation of the infectious protein isoforms.

As the brain continues to be studied and the systems within it are uncovered and researched, more potential treatment options for NDs become available. One such is the usage of the glymphatic system in AD in order to clear out Aβ and p-tau as the proteins accumulate. When the system was first described in 2012 by Maiken Nedergaard and colleagues, the dependency on astroglial cells was once again highlighted [86]. While most of the research on the glymphatic system has focused on the Aβ protein, such as in Peng et al., there is a growing number of studies focusing on the MT-associated tau protein, such as in Harrison et al. [53,87].

The App^NL-G-F/NL-G-F^ mouse model developed by Sakakibara and colleagues poses the potential to serve as a good base for developing therapeutic treatments for AD-related Aβ plaque formations [88]. Beyond just developing new animal models with better human accuracy, however, there needs to be a way to trace the presence of the proteins within humans, such as by developing ways to flag the proteins, similar to what Hosokawa-Muto, J. et al. did during a fluorescence resonance energy transfer (FRET) analysis on the protein, although this method was used in an attempt to elucidate structural information and required plasmid transfection of the gene coding for the fluorescent-labeled PrP [89].

### 6.2. Notable Advancements in Treatments in Recent Years

PrP^C^ can serve in a neuroprotective capacity when interacting with AβO in astrocyte cells to prevent oxidative damage that can eventually contribute to neurodegeneration [65]. However, PrP^C^ can also interact with subsets of AβO in AD to further cognitive impairment as the disease progresses [90]. Furthermore, differing biomarkers (especially Aβ) that are used in the diagnosis and monitoring of AD are also a field of increasing focus, such as monitoring the accumulation of neurotoxic protein aggregates and the resulting neurodegeneration through blood Aβ levels [91] or the potential for diagnosis and monitoring disease progression through the retina [92]. Furthermore, brain-derived tau has recently shown promise as another potential AD biomarker, outperforming the previously less-specific blood total-tau levels [93].

As reviewed by Murakami, K. et al., aptamers have become a popular topic in regard to their potential for treating amyloidogenic proteins, such as PrP^Sc^ and Aβ. The use of aptamers for treatment is a difficult but potentially prosperous possibility [94]. This is due to the existence of DNA and RNA aptamers in order to target a given part of a protein. clustered regularly interspaced short palindromic repeats (CRISPR)/Cas9 gene editing may also serve as a way to treat prions and prion-like proteins, especially for those with a genetic predisposition for a given disease. One study by Castle et al. in 2022 attempted to introduce a null Prnp allele or edited Prnp gene into mice, which, while overall unsuccessful, showed promising aspects for a basis to further develop this possibility by reworking the issues that arose within the study, such as focusing on developing a complete germline [95].

Autophagy stimulation is another treatment method that has been of popular interest in regard to treating prion infection due to the ability to manipulate autophagy at different points in the infection stages. This, along with other treatments, poses difficulties due to the fear of the likelihood of prions developing resistance to treatment. As such, one group has recently tried a combination of autophagy stimulation and cellulose ethers, as both are known to show positive results in regard to prion reduction, but this combination did not show as good results as compared to individual treatments, leaving still more to investigate for possible combination treatments [96].

Finally, vaccination treatment shows promise in reducing levels of protein aggregation. Vaccines against neurodegenerative diseases are not a new concept; however, early trials of vaccination treatment have shown adverse effects [97]. Recently, due to COVID-19, there has been growing scientific interest in DNA vaccines. These ideas have been applied to Aβ vaccination against Alzheimer’s disease, which has been shown to be potentially safer than traditional vaccines [97]. Additionally, traditional vaccines against aggregated alpha-synuclein have shown potential in combating Parkinson’s disease, with the PD01A vaccine recently completing phase I trials [97]. Unfortunately, while these vaccination treatments do show promise, this research is still new, and some adverse vaccine-related effects may still remain [97,98].

## 7. Conclusions

The significance of this review lies in improving our understanding of the mechanisms of structural changes and protein aggregation in prions and prion-like proteins that are seen in AD and PD and the neurodegeneration associated with each respective protein. As interest in prions and infectious proteins grows, the exploration of parallels and structural information will hopefully aid in developing increasingly successful treatments for preventing neurodegeneration before it starts, along with helping to prevent continued ongoing neurodegeneration from occurring.

It is becoming more and more apparent that neurodegenerative disease progression is somehow linked with the structures of the associated prion-like proteins. Therefore, as more information regarding the structure of the infectious prion protein is achieved and understood, it will hopefully lead to better treatments for diseases associated with prions and prion-like proteins, as explored throughout this review. Recent research on prions has begun to link novel structures and mutations, such as amino acid replacements, protein seeding, environmental changes, and hydrophobic cores, in prions and prion-like proteins to dysregulated homeostasis, leading to misfolding, propagation, and aggregation of these proteins. By summarizing recent advances in causal relations of structural anomalies and the potential of the aggregation model, we disclose underlying mechanisms in the onset of neurodegeneration from the perspective of prion diseases, AD, and PD and the possible interlinking of the proteins associated. Finally, by understanding the molecular background of these proteins, we can begin to explore novel treatment plans, such as aptimer and vaccine treatments.
